# Unklare Schwellung und Rötung nach einer Klavikulafraktur

**DOI:** 10.1007/s00104-022-01805-6

**Published:** 2023-01-26

**Authors:** U. Barth, D. Granowski, S. Stephan-Falkenau, P. Bönicke, M. Lehmann, F. Meyer

**Affiliations:** 1 Arbeitsbereich Gefäßchirurgie, Klinik für Allgemein‑, Gefäß- und Viszeralchirurgie, Helios Klinik Jerichower Land, Burg, Deutschland; 2 Institut für Gewebediagnostik/Pathologie, MVZ am Helios Klinikum Emil von Behring, Berlin-Zehlendorf, Deutschland; 3 Klinik für Radiologie, Helios Bördeklinik GmbH, Oschersleben, Deutschland; 4 Klinik für Allgemein‑, Viszeral‑, Gefäß- und Transplantationschirurgie, Universitätsklinikum Magdeburg A. ö. R., Otto-von-Guericke-Universität, Magdeburg, Deutschland

## Anamnese

Eine 57-jährige Patientin erlitt 4 Monate vor der gefäßchirurgischen Vorstellung ein Sturztrauma auf die linke Schulter. Eine zeitnahe ärztliche Vorstellung der Patientin erfolgte aber nicht. Im Verlauf kam es jedoch zur Entwicklung eines pulsierenden Tumors des linksklavikulären Bereiches mit Ausbildung eines zunehmenden Stauungsödems des linken Arms, venöser subkutaner Umgehungskreisläufe und einer Dermatitis des linken Armes.

Weitere wichtige Komorbiditäten umfassten einen insulinpflichtigen Diabetes mellitus und eine arterielle Hypertonie.

## Klinischer Befund

Im Bereich der linken Schulterregion imponierte eine ca. 20 × 20 cm große pulsierende Schwellung, die gerötet und überwärmt war. Subkutan zeichnet sich ein Venennetz von Umgehungskreisläufen ab. Zudem war der linke Arm geschwollen und im Sinne einer Stauungsdermatitis gerötet. Im medialen Bereich der Raumforderung ließ sich eine spitze Resistenz tasten. Die Untersuchung der peripheren Pulse ergab keine Defizite.

## Aufnahmelabor.

Das Aufnahmelabor ergab neben einem minimal erhöhten CRP-Wert (9,7 mg/l), einen minimal erhöhten TSH-Wert (4,34 mU/ml) ohne Erniedrigung der peripheren Schilddrüsenwerte im Sinne einer latenten Hyperthyreose und eine deutlich erhöhte Glutamyltransferase mit 3,14 µmol/ls. Die übrigen Werte lagen im Normbereich.

## Bildgebung.

**Figure 1:**
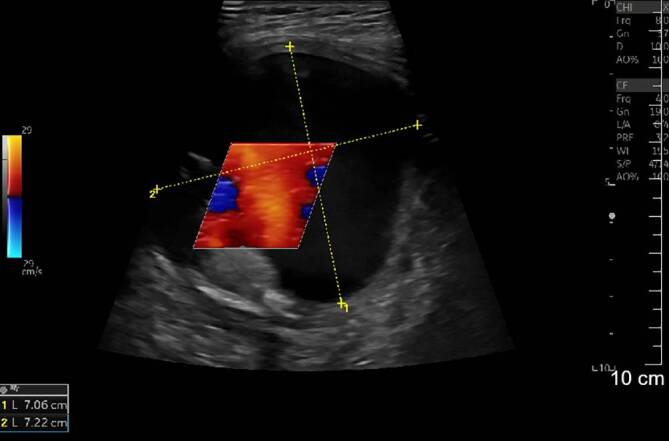

**Figure 2:**
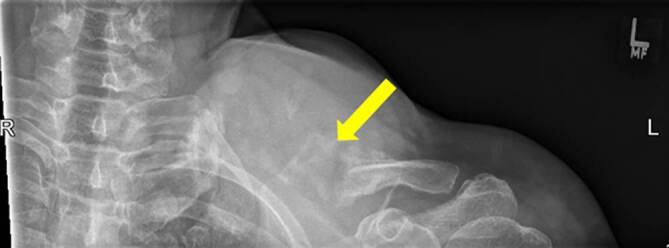

**Figure 3:**
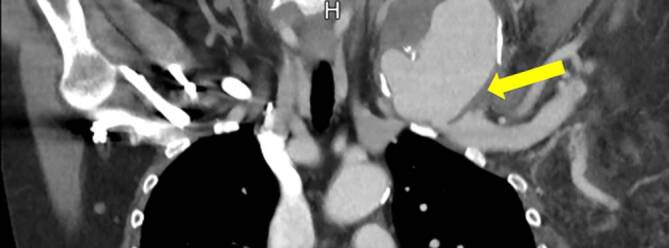

1. Die initiale Sonographie zeigte eine 7,1 × 7,2 cm echoleere Raumforderung mit echoreichen Thrombusformationen in der farbkodierten Duplexsonographie mit einer pulssynchronen vollen Farbfüllung (Abb. 1).
2. Die konventionelle Nativröntgenuntersuchung der linken Schulterregion dokumentierte einen ausgedehnten Defekt der Klavikula im mittleren Drittel (Abb. 2).
3. Die nachgeschaltete CT-Angiographie (CTA) zeigte im Bereich der linken Schulter eine ca. 8,6 × 7 cm große perfundierte Raumforderung, entspringend aus der A. subclavia sinistra, welche die Umgebungsstrukturen verdrängt (Abb. 3).

## Wie lautet Ihre Diagnose?

### Therapieentscheidung.

Aufgrund der ausgeprägten Umgebungsreaktion wurde die Indikation zur offenen arteriellen Rekonstruktion im A.-subclavia-Stromgebiet links mit infraklavikulärem Zugang gestellt.

### Therapie.

**Figure 4:**
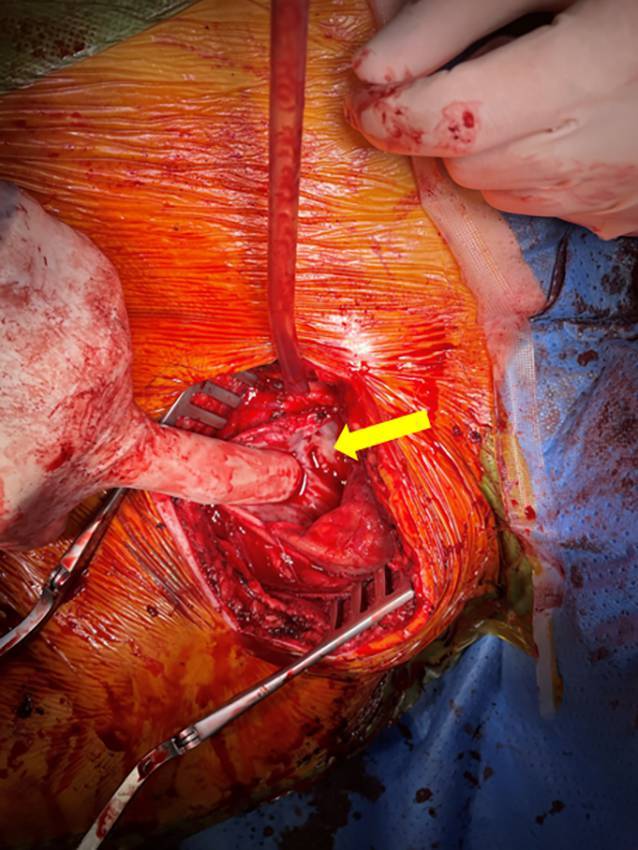

*Operativ*: Es erfolgte die Freilegung des durch eine kräftige Kapsel geschützten Pseudoaneurysmas, Eröffnung desselben und Entfernung von Hämatom und Thrombussaum. Die Kapsel wurde zu zwei Drittel reseziert und nach Darstellung der Perforationsstelle erfolgte der Direktverschluss mittels Übernähung durch eine fortlaufende Naht mit nichtresorbierbarem Nahtmaterial. Des Weiteren erfolgte die Entfernung des ursächlichen spitzen Knochenendes der Klavikula (Abb. 4).

### Histopathologie.

**Figure 5:**
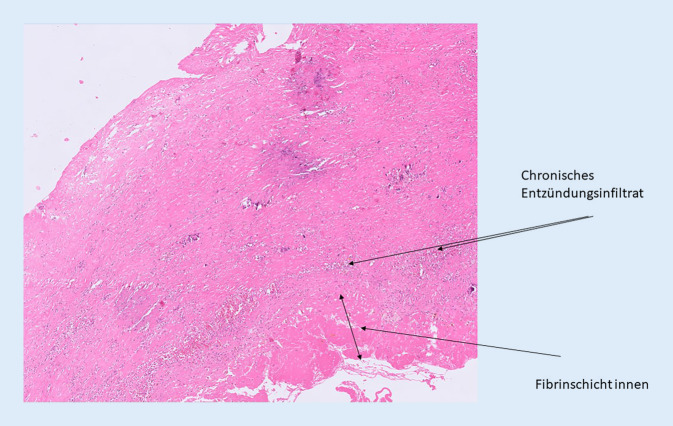

Die Pseudoaneurysmawandanteile wurden als solche bestätigt, charakterisiert durch Fibrose, Blutungsresiduen, granulierende Reaktion, nach luminal hin kautschukhyalinem Thrombus mit Verkalkung. Des Weiteren wurde ein knöchernes Fragment begutachtet, welches Geflechtknochenbildung und eine fokal osteoklastische Reaktion, Blutungsresiduen und eine ausgeprägte narbige Fibrose in der Umgebung einschließlich granulierender Reaktion zeigte (Abb. 5).

### Weiteres Prozedere.

Aufgrund des ausgeprägten Defektes der Klavikula von etwa 6 cm war eine traumatologische oder plastische Rekonstruktion des Knochens nicht sinnvoll, zumal die Patientin keine wesentliche Einschränkung ihrer normalen häuslichen Tätigkeit und ihrer Lebensqualität verspürte.

### Definition.

Bei einem Aneurysma spurium, falschem Aneurysma oder Pseudoaneurysma liegt ein Defekt aller 3 Schichten der Gefäßwand vor, worauf das Blut aus dem Gefäß austritt und von einer bindegewebigen Kapsel umgeben wird [1].

### Differenzialdiagnosen.

Zu den Differenzialdiagnosen sind neben Hämatom, Serom, Abszess auch eine Tumorläsion (Pancoast-Tumor), ein Lymphknotenpaket, Osteom oder Osteomyelitisherd zu zählen.

### Vorkommen/Häufigkeit.

Pseudoaneurysmen der A. subclavia als Folge einer Klavikulafraktur sind seltene Ausnahmen, die in der wissenschaftlichen Literatur nur in fachspezifischen Kasuistiken aufgearbeitet, dokumentiert und eingeordnet sind.

### Etiologie – prädestinierende Faktoren.

Die häufigste Ursache für Verletzungen der A. subclavia sind die penetrierende Gefäßläsionen – stumpfe Traumata sind nur für 1–5 % der Verletzungen der A. subclavia verantwortlich und sind in der Regel mit Frakturen oder Dislokationen verbunden [2].

### Diagnostik.

Die klinische Untersuchung des Lokalbefundes ergibt bereits durch den pulsierenden Charakter und das typische Schwirren, welches mit dem Stethoskop als typisches Fistelgeräusch auskultiert werden kann, erste Hinweise auf ein Aneurysma spurium.

Zur genaueren Diagnostik eines Pseudoaneurysmas der A. subclavia ist eine Kombination bildgebender Verfahren sinnvoll, wobei Maskanakis et al. [3] in ihrem systematischen Review eine anfängliche CT/CTA zusammen mit einer prozeduralen DSA in der überwiegenden Mehrheit der eingeschlossenen Studien fanden. Zur primären Sicherung einer Klavikulafraktur kommt in erster Linie eine Nativröntgenaufnahme zur Anwendung. Bei klinischem Verdacht eines Pseudoaneurysmas sollte sich eine leicht durchführbare Sonographie und farbkodierte Duplexsonographie anschließen, die einen schnellen Überblick über eine pulsierende Raumforderung im Klavikulabereich ergibt. Die Kombination einer CTA und DSA erlaubt eine genaue Beurteilung der Ausdehnung des Pseudoaneurysmas, der umgebenden oder verdrängten Strukturen, eine genaue Lokalisation der Perforationsstelle bzw. exakte Einstufung der Art der Läsion der betroffenen Arterie und eine Beurteilung der Fraktur und Frakturenden zur Planung einer Klavikularekonstruktion.

### Therapieoptionen.

Zur operativen Versorgung ergeben sich mehrere Therapiemöglichkeiten. Aufgrund der zunehmenden Verfügbarkeit, guten technischen Handhabung und der hohen technischen Erfolgsquote als auch des eher limitierten Interventionstraumas hat sich eine endovaskuläre Versorgung weitgehend durchgesetzt. Weitere Vorteile bieten die Zugangswege über die A. brachialis oder A. femoralis communis durch eine geringe Invasivität sowie deren Kombinationsmöglichkeiten [3]. Bei der Verwendung gecoverter Stents wird in der Regel eine sichere Abdeckung der Perforationsstelle erreicht, eine Kombination mit Coils oder Klebematerialien ist in der Regel nicht nötig.

Die offenen operativen Techniken bieten den Vorteil der lokalen Resektion des Pseudoaneurysmas und damit Behandlung der raumfordernden Wirkung, die neben den Stauungssymptomen durch Kompression des venösen Abflusses ebenfalls zu neurologischen Störungen im Bereich des Armes durch Alteration der Plexusstrukturen führen können. Im Vergleich zur endovaskulären Methode sind natürlich die operationsbedingten Komplikationsmöglichkeiten ins Feld zu führen. So sind Verletzungen des Plexus brachialis und der V. subclavia mit einer höheren Morbidität behaftet und unbedingt zu vermeiden. In dem verbliebenen Raum des Pseudoaneurysmas ist die Bildung eines Seroms trotz Drainage möglich.

**Diagnose:** Klavikulafrakturinduzierte arterielle Gefäßalteration mit massivem Aneurysma spurium der A. subclavia sinistra und Umgebungskompression der V. subclavia und venöser Abflussstörung im linken Arm mit begleitender Stauungsdermatitis

Die Verletzung der subklavialen Gefäße ist selten, da sie durch den Musculus subclavius, das Schlüsselbein, die erste Rippe und die tiefe zervikale Faszie geschützt sind. Etwa die Hälfte der Verletzungen der A. subclavia ist auf die Dislokation des proximalen Teils der Klavikula durch den Zug des Musculus sternocleidomastoideus nach oben zurückzuführen [4]. Pseudoaneurysmen der A. subclavia können lange Zeit asymptomatisch bleiben. Häufige Symptome sind Schmerzen in der Brust, im Rücken und/oder im Arm, Horner-Syndrom, Heiserkeit der Stimme, Dysphagie, Armischämie, distale Embolie, Ruptur und venöse Stauung im Arm aufgrund des Drucks des Aneurysmas [3].

## Fazit für die Praxis

Ein großes Pseudoaneurysma der A. subclavia mit Osteolyse der Klavikula im mittleren Drittel als Folge einer Fraktur ist eine Rarität, die einer interdisziplinären gefäß-/traumatologisch-/plastisch-chirurgischen Behandlung bedarf. Durch die zunehmende Etablierung und sichere Handhabung der endovaskulären Techniken ist die offen-gefäßchirurgische Therapie in den Hintergrund getreten. Jedoch bietet die offene gefäßchirurgische Rekonstruktion im individuellen Fall auch Vorteile, gerade wenn Kompressionserscheinungen durch die schiere Größe des Aneurysmas zu vaskulären und nervalen Komplikationen führen.
